# Impact of the Surgical Environment on the Incidence, Timing, and Severity of Complications Associated With Oromaxillofacial Oncologic Surgery in Dogs

**DOI:** 10.3389/fvets.2021.760642

**Published:** 2021-12-17

**Authors:** Brittney E. Rigby, Kevin Malott, Susannah J. Sample, Scott J. Hetzel, Jason W. Soukup

**Affiliations:** ^1^Department of Surgical Sciences, School of Veterinary Medicine, University of Wisconsin, Madison, WI, United States; ^2^Department of Biostatistics and Medical Informatics, University of Wisconsin, Madison, WI, United States

**Keywords:** oromaxillofacial surgery, surgical complications, oncologic surgery, dog, surgical environment

## Abstract

Numerous reports describe complication rates associated with oromaxillofacial oncologic surgery in dogs, however, investigation regarding the impact of the surgical environment on the incidence of complications is under reported. The objective of this retrospective cohort study, including 226 dogs surgically treated for oromaxillofacial tumors between January 1, 1997 and December 31, 2018, is to evaluate the impact of the surgical environment on the incidence of complications in oromaxillofacial oncologic surgery in dogs. A secondary objective is to report the incidence of local complications in oromaxillofacial oncologic surgery and characterize the type, timing, and severity of complications encountered. Incidence of complications was identified to be 69.9%. No significant association was identified between the incidence, timing, or severity of complications and the training background of the clinician, physical location of the procedure, or the ostectomy instrument used. These results suggest that the surgical environment has little impact on the incidence, timing, and severity of complications in dogs undergoing oromaxillofacial oncologic surgery. The results also emphasize the importance of preparing the surgical team and the client for a high incidence of complications associated with oromaxillofacial oncologic surgery in dogs and indicate that both short-term and long-term follow up is important in these cases. Oromaxillofacial surgery performed by residents-in-training within a veterinary teaching environment with adequate supervision appears to be safe.

## Introduction

Numerous studies have documented the complications and outcomes related to oromaxillofacial oncologic surgery in dogs (1–11). Complication rates associated with maxillectomy in the dog have been reported as 0–53.4% (1, 2, 4, 5, 12), while the complication rate following mandibulectomy has been reported as 12–44% (6, 7, 9). These studies considered the effect of several patient-dependent variables on the complication rate including patient signalment, anatomical tumor location, tumor type, tumor stage, histologic margin assessment, and surgical approach. One of these reports included the instrument used for ostectomy as a potential variable related to complication outcome and found no association between bone cutting instrument and requirement for an intraoperative blood transfusion (12). Antibiotic protocol has been suggested as a factor in the prevention of surgical site infection although this suggestion is not strongly supported by evidence in the veterinary literature (13–16). The antibiotic protocol selected is considered a patient-dependent variable, as the contamination level of the surgery and patient comorbidities influence antibiotic choice and duration of use. Anesthetic variables, such as the use of propofol, time under anesthesia, and surgical time are considered to be significant contributors to peri-and-post-operative complications (13, 14, 16). These factors are also considered to be patient-dependent as the anatomical location and size of the tumor would dictate the surgical and anesthetic time and the choice of anesthetic drugs is often determined by the patient's temperament, age, and comorbidities.

There has been no other published investigation regarding how patient-independent variables impact the rate of complication associated with oromaxillofacial oncologic surgery in dogs. The authors have termed these patient-independent variables the “surgical environment” which, for the purposes of this study, includes the training program of the clinician (American College of Veterinary Surgeons [ACVS] or American Veterinary Dental College [AVDC]), the experience of the clinician (residency-trained faculty or residents-in-training), the surgical instrument(s) used for ostectomy, and the physical location in which the surgery was performed (surgical operatory or dental operatory).

The primary objective of this study was to assess how the surgical environment impacts the complication rate of oromaxillofacial oncologic surgery in dogs. A secondary objective was to report the incidence of complications in this population and describe the various types, timing, and severity of complications encountered. Our hypothesis was that the surgical environment would have little impact on the complication rate associated with these procedures and that the incidence of complications would be similar to previously reported data.

## Materials and Methods

Medical records of patients that underwent maxillofacial surgery at this institution between January 1, 1997 and December 31, 2018 were identified by using the following search terms: mandibulectomy, maxillectomy, zygomectomy, coronoidectomy, oronasal fistula, cleft palate, orbitectomy, incisivectomy, piezosurgery, condylectomy, rim excision, and nasal planectomy. Using these terms, 880 patients were identified. Further review of these cases was performed to identify those patients that underwent surgical treatment for oncologic reasons. The majority of the identified 880 cases were excluded because they did not have oncologic disease, the recommended surgery was declined, or the procedure was discussed but not recommended. Three hundred and seventy-five cases were identified. For case standardization, only those cases which had pre-operative head computed tomography (CT) were included. Additionally, case inclusion required access to the patient's medical history, physical examination at the time of the surgery, surgical report, hospitalization summary, anesthetic record, and tumor histology report. Patients that were treated with palliative-intent surgery, rather than curative intent surgery, were excluded. Surgeries involving facial soft tissues without ostectomy were excluded. Finally, a description of a post-surgical recheck at this institution or with the referring veterinarian at least seven days after the surgical procedure was required. Table 1 details the variety of oromaxillofacial surgical procedures that were included in this analysis. Due to the wide variety of surgical procedures included, the anesthetic time for each procedure was evaluated as a contributor to the incidence of complication as an indirect assessment of the difficulty of the procedure being performed.

**Table 1:** Description of the type of surgical procedures performed and number of each procedure performed.

**Surgical Procedure**	**Surgical Operatory**	**Dental Operatory**
Incisivectomy	2	5
Incisivectomy with nasal planectomy	3	1
Bilateral rostral maxillectomy with nasal planectomy	3	1
Caudal maxillectomy	12	5
Rostral maxillectomy	8	5
Caudal maxillectomy including zygoma +/− orbital bones	13	4
Bilateral rostral maxillectomy	8	10
Orbitectomy with enucleation	1	0
Central maxillectomy	17	15
Caudal maxillectomy, orbitectomy, mandibular coronoidectomy	1	1
Rostral bilateral mandibulectomy	15	36
Rostral unilateral mandibulectomy	2	4
Segmental mandibulectomy	14	10
Caudal mandibulectomy	6	4
Total mandibulectomy	11	2
Mandibular rim excision	2	3
Subtotal mandibulectomy	2	2

Surgical complications were identified and analyzed. Complications unrelated to the surgical procedure were excluded (i.e., diarrhea, regurgitation, esophagitis, etc.). Each complication was classified as catastrophic, major, or minor based on the definitions of surgical complications previously proposed by Cook et al. (17). A catastrophic complication was defined as that which resulted in “permanent unacceptable function,” death, or was the cause for euthanasia. Major complications were those which required professional intervention. Major complications were further divided into those requiring surgical treatment or medical treatment (major-surgical and major-medical, respectively). For example, if dehiscence of a surgical site resulted in the formation of an oronasal fistula requiring surgical closure of the defect, this was classified as a major-surgical. If the same patient also received additional pain medications for the described complication, the categorization of the severity remained the same (major-surgical) and was not counted again as a major-medical complication. Conversely, if dehiscence of a surgical site resulted in adequate healing by second intension and only supportive antimicrobial and pain medications were prescribed, this was classified as a major-medical. Minor complications were defined as those which did not require any additional medical or surgical treatment. Examples of minor complications include tongue protrusion, epistaxis, and self-resolving sialoceles. Each complication had the potential for varying levels of severity. For example, hemorrhage may have been classified as major-surgical if surgical intervention was required, major-medical if transfusion of blood products was required, or classified as minor if it was noted in the medical record but did not require surgical or medical intervention. Hemorrhage requiring surgical intervention and medical intervention was classified as major-surgical, because complications at each time point were counted as the most severe category only. These classifications were based on the clinician's recommendations as recorded in the medical record; whether or not the client was compliant to those recommendations was not considered in the analysis.

Complications were also analyzed based on the time frame in which they were identified. Immediate complications were defined as those which were identified peri-operatively or up to 24 h after surgery. Short-term complications were defined as those identified between 25 h and 30 days post-operatively. Long-term complications were those identified >30 days following the procedure. For those patients with multiple complications at a single time point, only the complication with the highest severity was included in the statistical analysis. Complications that were identified at one time point and persisted into a longer time period despite treatment were described as two separate complications. However, a complication identified at one time point that was persistent due to lack of treatment was not considered a complication at later time points. The statistical analysis only included the most severe complication for any given patient at each time point. For example, a patient who was reported to have untreated epistaxis and facial nerve paralysis within the first 24 h following surgery was counted as a patient with immediate minor complications. If the same patient presented 14-days post-operatively with persistent epistaxis and dehiscence requiring surgical closure of the defect, antibiotics, and pain medications, they were only counted as a patient with a short-term major-surgical complication.

The nature of the training program and the experience of the primary clinician was considered as a variable in this analysis. Surgeries were either performed under the care an ACVS trained veterinarian (residency trained faculty or resident-in-training veterinarian) or an AVDC trained veterinarian (residency trained faculty or resident-in-training veterinarian). The physical location in which the surgery was performed was extracted from the medical records. Procedures were performed in either a surgical operatory or a dental operatory. Finally, the surgical instrument used for ostectomy was considered. Ostectomy instruments were categorized into four groups: manual instruments (osteotome and mallet), electric and air-driven surgical units (oscillating saw, sagittal saw, reciprocal saw, etc.), air-driven dental units (high-speed hand-piece with various cutting burs), and piezoelectric surgical units.

Statistical analysis evaluated the relationship between the variables and complications using univariable logistical regression models. Results of the logistic regression models are presented as odds ratios (OR) with 95% confidence intervals (CI) and *p*-values. All tests were conducted at a standard 5% significance level. Statistical calculations were made with R version 3.5 software. Over the 21-year period that these medical records were analyzed, there were various changes in the hospital staff, surgical equipment, and general medical and surgical knowledge. The skill level and complexity of procedures performed by any individual clinician was likely to have changed over time as well. To account for these intricate changes, a logistical regression model controlling for the surgical date (by year) was used.

## Results

Two hundred and twenty-six dogs met the inclusion criteria and were included in this report. The population included 2 (0.9%) intact females, 103 (45.6%) spayed females, 15 (6.6%) intact males, and 106 (47%) castrated male dogs. The mean weight was 29.2 kg (3.4–71 kg) and the mean age was 8.2 years old (7 months−16 years old). Patient signalment was not significantly associated with surgical complications.

A total of 301 complications were described in 167 out of 226 (69.9%) patients. There was a wide variety of surgical procedures performed, ranging from mandibular rim excision or incisivectomy for treatment of benign tumors, to excision of highly invasive malignant tumors with en bloc excision of the orbitozygomaticomaxillary complex and regional lymph node extirpation. The types of surgical procedures included are detailed in Table 1. The length of the procedure (i.e., anesthetic time) was used to estimate the relative difficulty of each procedure. The incidence of overall complication rate as related to anesthetic time was not statistically significant (*p* = 0.511).

Many patients had multiple complications at more than one time point. Statistical analysis included only the most severe complication for each individual patient at each time point, resulting in a total of 238 complications analyzed in this cohort. Table 2 summarizes all the surgical complications described in this dataset. Two patients had immediate catastrophic complications resulting in death. The cause of death in both cases was determined to be related to the anesthetic event or the patient's comorbidities and thus not included in the statistical analysis. No short-term or long-term catastrophic complications were identified. Sixty-nine (30.5%) patients had immediate complications, 117 (51.8%) patients had short-term complications, and 52 (23.0%) patients had long-term complications. Seventy-three (32.3%) patients had major-surgical complications, of which four (5.5%) were identified in the immediate time period, 42 (57.5%) were short-term, and 27 (37.0%) were long-term complications. Sixty-three (27.9%) patients had major-medical complications, of which 30 (47.6%) were identified in the immediate time period, 28 (44.4%) were short-term, and 5 (7.9%) were long-term complications. One hundred and two (45.1%) patients had minor complications requiring no additional treatment, of which 35 (34.3%) were identified in the immediate time period, 47 (46.1%) were short-term, and 20 (19.6%) were long-term complications. The timing and severity of complications is summarized in Table 3.

**Table 2:** Description of all complications (*n* = 301) by time of identification.

	**Number of complications**		
**Complication**	**Immediate**	**Short-term**	**Long-term**
Abscess formation at surgical site	-	-	1
Anemia	1	-	-
Bacterial rhinitis	-	-	2
Bony sequestrum	-	1	-
Buccal/labial mucosal ulcerations	-	3	1
Cellulitis	-	1	-
Dehiscence	1	55	4
Dermatitis	1	4	2
Difficulty prehending food or grasping toys	-	8	-
Dry eye	-	1	-
Edema	1	1	-
Emphysema	2	-	-
Enophthalmia	-	1	-
Epistaxis	23	1	-
Facial nerve paralysis	2	1	2
Halitosis	-	3	-
Hemorrhage	34	-	-
Iatrogenic jaw fracture	1	-	-
Iatrogenic trauma to adjacent teeth	1	-	-
Intractable pain	-	2	-
Intrinsically stained or non-vital tooth or teeth	-	2	6
Labial entrapment with or without ulceration	-	11	1
Limited range of motion of the temporomandibular joint	-	-	2
Mandibular drift	4	18	8
Masticatory myositis	-	1	-
Oronasal fistula	-	22	9
Periodontal compromise to adjacent tooth or teeth	-	2	2
Ptyalism	-	3	1
Pyrexia	0	1	-
Seroma	4	-	-
Sialocele	13	4	-
Subcutaneous swelling	1	-	-
Surgical site infection	-	17	-
Surgical site swelling	-	3	1
Temporomandibular joint pain	-	1	1
Tissue necrosis at surgical site	-	1	-
Traumatic malocclusion	-	1	-

**Table 3:** The number of most severe complication per patient (*n* = 238) and percentage of total patients (*n* = 226) categorized by severity and timing.

	**Immediate**	**Short-term**	**Long-term**	**Total complications**
Catastrophic	0 (0.0%)	0 (0.0%)	0 (0.0%)	0 (0.0%)
Major–Surgical	4 (1.7%)	42 (18.6%)	27 (11.9%)	73 (32.3%)
Major–Medical	30 (13.3%)	28 (12.4%)	5 (2.2%)	63 (27.9%)
Minor	35 (15.5%)	47 (20.8%)	20 (8.8%)	102 (45.1%)
Total complications by time	69 (30.5%)	117 (51.8%)	52 (23.0%)	238

*Immediate time frame, peri-operative to 24 h post-operative; Short-term time frame, 25 h to 30 days post-operative; Long-term time frame, >30 days post-operative*.

One hundred and twenty-seven (56.2%) patients were operated under the care of an AVDC trained clinician/resident and 98 (43.4%) patients by an ACVS trained clinician/resident. One patient (0.4%) was operated on with combined efforts from AVDC and ACVS clinicians. The difference in overall complication rate between the training groups was not statistically significant (*p* = 0.323). Incidence of immediate, short-term, and long-term complications were not statistically significant between training groups.

Within this population, the experience of the primary clinician was clearly identified in 74 (32.7%) medical records. Of these 74 cases, 37 (50%) were performed by a resident-in-training and 37 (50%) were performed by residency trained faculty clinicians (AVDC, ACVS). Twenty-two out of 37 (59.5%) surgeries performed by residents-in-training developed complications while 28/37 (75.7%) surgeries performed by faculty clinicians developed complications. This difference was not statistically significant (*p* = 0.109). When timing of the complications was considered, faculty clinicians had twice as many complications in the immediate time period with 13/37 (35.1%) patients developing immediate complications in comparison to 6/37 (16.2%) of the surgeries performed by residents-in-training, however, this was not statistically significant (*p* = 0.054). Complications at other time points were not significantly different between groups. Short-term complications were reported in 19/37(51.4%) surgeries performed by faculty clinicians and 17/37 (45.9%) surgeries performed by residents-in-training (*p* = 0.571). Long term complications were reported in 9/37 (24.3%) cases performed by faculty clinicians and 9/37 (24.3%) cases performed by residents-in-training (*p* = 0.863).

The surgical instrument used for ostectomy was not significantly associated with the incidence or timing of reported complications (*p* = 0.308). The surgical ostectomy instrument was recorded in 207 (91.6%) of the total 226 cases. Manual instruments were used in 24 (11.6%) cases with 15 (62.5%) of those developing complications. Air-driven and electric surgical units were used in 57 (27.5%) cases with 37 (64.9%) developing complications. Air-driven dental ostectomy instruments were used in 35 (16.9%) cases with 22 (62.8%) developing complications. A piezoelectric surgical unit was used in 21 (10.1%) cases, of which 19 (90.5%) had complications reported. A combination of ostectomy instruments were used in 70 (33.8%) cases with 49 (70.0%) developing complications.

One hundred and twenty (53.1%) cases were performed in a surgical operatory and 106 (46.9%) were performed in a dental operatory. There was no significant association between the physical location of the procedure and the incidence or timing of complications. Table 4 summarizes the incidence of total complications as impacted by the components of the surgical environment.

**Table 4:** The number of dogs (*n* = 226) which developed complications as categorized by the components of the defined surgical environment.

**Variable**	**Total # of dogs with no complications (%)**	**Total # of dogs with complications (%)**	***p*-value**
Background Training^*^			
ACVS	36 (16.0)	62 (27.6)	0.323
AVDC	32 (14.2)	95 (42.2)	—
Experience of primary clinician^**^			
Faculty clinicians	9 (12.2)	28 (37.8)	0.109
Resident in training	15 (20.3)	22 (29.7%)	—
**Physical location of Surgery**			
Dental suite	31 (13.7)	75 (33.2)	0.6
Surgical operatory	37 (16.4)	83 (36.7)	—
Ostectomy instrument^***^			
Surgical unit (air-driven or electric)	20 (9.7)	37 (17.9)	0.308
Dental unit (air-driven)	13 (6.3)	22 (10.6)	—
Manual	9 (4.3)	15 (7.2)	—
Piezoelectric surgical unit	2 (1.0)	19 (9.2)	—
Combination of instrumentation	21 (10.1)	49 (23.7)	—

* *n = 225, one patient had surgery performed with ACVS and AVDC clinicians and was not included in this statistical analysis;*

** *n = 74, the primary clinician was identified in only 74/226 patients;*

*** *n = 207, the ostectomy instrument was recorded in only 207/226 medical records*.

## Discussion

The present study investigates the impact of variables which are independent of the specific patient or tumor in the development of complications in dogs undergoing oromaxillofacial oncologic surgical procedures. The findings of the present study support the hypothesis that the surgical environment, as defined within this report, has little impact on the complication rate. For the purposes of this report, these patient-independent variables are considered the “surgical environment” and include the physical location in which the surgery was performed, the background and experience of the clinician, and the instrument used for ostectomy. The overall incidence of complications in this cohort is higher than that previously reported (1, 2, 4–7, 10, 12). The high incidence of overall complications in this cohort may be related to the wide range of included surgical procedures under the umbrella of oromaxillofacial oncologic surgeries. Surgical procedures in this population included everything from incisivectomy to excision of the entire orbitozygomaticomaxillary complex. Interestingly, the reported incidence of complications in this cohort at each given time point (immediate, short-term, long-term) are more consistent with previous literature. It may be that the higher overall complication incidence reported here is due to differences in the inclusion criteria and characterization of the timing of reported complications as compared to previous studies. This report also included a larger population than many of the cited articles, some of which included very few subjects. Finally, we reported complications that did not require any specific treatment, such as ptyalism, which may have been overlooked in other reports as these could be considered by some to be expected consequences rather than complications. While these types of complications may have a low impact on a patient's quality of life, they may be perceived by owners as notable.

As suggested by Cook et al. to describe orthopedic procedures in veterinary medicine, complications were defined as catastrophic, major, or minor (17). Major complications were further categorized as either requiring surgical or medical treatment. Complications were also characterized by the timing in which they presented after surgery (immediate, short-term and long-term). Although all patients had a least one recheck appointment, one of the limitations of this analysis is that many of the patients were not examined during all three time points. The time of a complication was categorized by the date on which the complication was recognized and described in the medical record, not necessarily the time at which the complication truly occurred. For example, an oronasal fistula that occurred 12 days post-operatively but was not identified until a recheck appointment 32 days post-operatively would have been categorized as a long-term complication. This may have resulted in misrepresentation of the timing of complications, and is an inherit limitation associated with the retrospective nature of this study.

One-third of the patients had immediate complications. Two of these patients suffered catastrophic complications resulting in death. Both patients had multiple confirmed and/or suspected comorbidities (laryngeal paralysis, uncharacterized cardiac and renal disease, hypothyroidism) that likely contributed to their deaths. Local surgical complications were not described to have contributed to the demise of these patients and therefore, they were excluded from the statistical analysis. Of the 69 complications that occurred immediately, four patients (5.8%) required surgical treatment and 30 (43.5%) required medical treatment. These findings suggest that close monitoring of a patient that has undergone oromaxillofacial oncologic surgery within the 24-h post-operative period is essential for early detection of treatable complications. Half (50.7%) of the immediate complications were minor and did not require any surgical or medical intervention.

Just over half (51.8%) of the patients experienced short-term complications. Of these patients, 35.9% had complications that necessitated a second surgical procedure (major-surgical) while 23.9% had complications that did not require additional treatment (minor). This is an important finding to consider when preparing a client for oromaxillofacial oncologic surgery with regards to the anticipated costs, expected duration of diet and activity restrictions, projected time commitment for recheck and follow-up appointments and procedures.

Long-term complications were reported in less than a quarter of the patients (23%). Over half of the patients (51.9%) who had long-term complications required surgical treatment (major-surgical). These findings suggest that a standard 10–14 day post-operative recheck may be inadequate at identifying major complications. Alternatively, as discussed above, despite these complications being identified >30 days post-surgery, the complication may have truly occurred earlier without detection or description in the medical record.

While it was a secondary objective of this publication to describe the reported complications, the reader must recognize that not every complication described here is anticipated or expected with all oromaxillofacial oncologic surgery. For example, mandibular drift was a commonly reported complication of mandibulectomy that often necessitated an additional procedure to alleviate self-trauma from the malocclusion. This complication would only be anticipated following specific mandibulectomy surgeries and would not be an expected complication following any maxillectomy procedure (18–20). A second example would be development of oronasal fistula following a maxillectomy. While this complication is one that was not uncommonly reported and typically requires a second surgical procedure, it would not be a complication expected following mandibulectomy procedures. Therefore, describing the incidence of specific complications was not undertaken in the context of this cohort. Additionally, incomplete margins on the tumor excision and tumor recurrence were not considered to be a surgical complication in this data set; the authors used the definition of a surgical complication suggested by Sokol and Wilson when considering which outcomes constitute a surgical complication (21). Using this definition, the presence of a tumor or recurrence of a tumor is not a direct result of the surgery itself.

Within this cohort, there was no significant association between the physical location of the procedure (surgical operatory *vs*. dental operatory) and the development or timing of complications. The dental operatory at this institution is used as both an exam room with conscious patients being examined throughout each day in addition to a surgical suite where a variety of dentistry and maxillofacial surgical procedures are performed. It is standard practice at this institution to use sterile draping and instrument technique when performing surgery in the dental operatory. The primary surgeon in the dental suite does not perform a surgical scrub, but is adorned with sterile gloves and a surgical cap. The assisting personnel are not routinely outfitted with traditional operating room attire. At this veterinary hospital, oromaxillofacial oncologic surgeries are performed in the dental operatory exclusively by AVDC faculty and resident-in-training clinicians while the surgical operatory rooms are used by both AVDC and ACVS practitioners. It is well-documented that the majority of surgical site infections, not just confined to the oral cavity, are related to the patient's own microflora, followed by transmission of bacteria from hospital staff to the patient; the sterility of the room in which the procedure is performed is considered to be the least likely cause of SSI (22–24). Furthermore, procedures using high speed ostectomy units and water lavage within the oral cavity result in aerosolization of oral bacteria and some degree of contamination of the surgical site regardless of the sterility of the room in which the procedure is performed. All surgery performed within the oral cavity has some degree of contamination regardless of the physical location in which the procedure is performed.

The ostectomy instrument had no significant impact on the development of complications. Factors affecting ostectomy efficiency, precision, safety, and post-ostectomy healing include: (1) the size of the instrument and instrument attachments, (2) speed of ostectomy device, (3) learning curve associated with use of the instrument, (4) water-cooling ability, and (5) effect of the instrument on adjacent soft tissues (25). Piezoelectric ostectomy is a relatively new technology that allows excision of bone without compromise to surrounding soft tissues using high-frequency vibration (26). This technology is reported to have high precision, little thermal damage to bone, reduced soft tissue trauma including reduced risk of damage to neurovascular structures, and improved visualization due to the combination of temporary cauterization of small vessels and continuous saline lavage (25–28). All of these things contribute toward an expected decrease in surgical complications. However, there is a reportedly steep learning curve to proper use of piezoelectric equipment and it is a slower means of ostectomy as compared to air-driven surgical and dental instruments (27, 29, 30). While not statistically significant, 19 out of 21 patients in which a piezoelectric saw was used had complications. This may be explained by the choice to use this type of surgical instrument for more complex surgeries or may be due to the steep learning curve described in other publications (25–28). In agreement with this study, Carrol et al. reported no significant association between the surgical bone cutting instrument used (high-speed hand-piece with rotary bur or oscillating bone saw) on the necessity for blood transfusion in patients undergoing a modified caudal maxillectomy procedure (12). The choice of surgical instrument is often based on which tools are available to the clinician and clinician's preference. However, there may be patient-dependent variables that guide a clinician's instrument choice. For example, a clinician may elect to use a piezoelectric unit for excision of tumors in the caudal maxilla due to the intricate vascular anatomy in that region. Conversely, the piezoelectric unit is a slower means of ostectomy and the clinician may elect to use an air-driven surgical unit for patients with high-anesthetic risk in order to reduce surgical time. The retrospective nature of the current study prohibits evaluation of a cause for specific instrument choice. The findings in this cohort indicate that selection of ostectomy instrument does not strongly influence the incidence of complications in oromaxillofacial oncologic surgery in dogs.

The background training (AVDC or AVCS) of the veterinarian performing the procedure did not have a significant impact on the development of complications. To the author's knowledge, there are no other studies that have examined outcomes of procedures performed by veterinarians with different training backgrounds. These findings suggest that the client and referring veterinarian should not expect any significant difference in the outcome of oromaxillofacial oncologic surgery when making the decision to seek treatment with an AVDC or ACVS veterinary practice. The likely more important patient-independent factor in predicting successful outcomes in oromaxillofacial surgery is the experience level of the supervising clinician.

When considering the influence of the clinician's experience on the incidence of complications, this data set revealed a near significant (*p* = 0.056) increase in the immediate complication rate between when faculty clinicians undertook procedures when compared to the resident-in-training veterinarians. This finding is most likely attributed to the teaching environment in which resident-in-training veterinarians are most often performing less technically advanced procedures and more likely to be assisting faculty clinicians with more advanced surgeries that would inherently be expected to have a higher rate of complications. Due to the low number of cases identified in this cohort that had a clearly defined primary clinician, the statistical significance found within the immediate time frame should be cautiously considered for relevancy. Most of the medical records examined did not clearly define the primary clinician associated with each case; during the period covered by this retrospective study, specific assignment of primary surgeons was not recorded in medical records at our hospital. Furthermore, when multiple surgeons were listed, it was unclear how much involvement each surgeon contributed to the procedure. This is a potential limitation of the data described in this report as the cases reported to have been performed primarily by a resident-in-training likely had differing levels of faculty supervision. There are various reports in the veterinary literature addressing the difference in surgical complications related to the experience of the clinician (31–33). These reports generally support the finding described here that the complication rate following surgical procedures is not significantly increased when performed by resident-in-training veterinarians. In these reports, it is again unclear the specific degree of involvement that faculty clinicians had in each procedure performed by a resident-in-training. There are numerous publications in the human literature addressing this query, as well. The majority of the literature suggests that supervised resident involvement in various surgical procedures, including high-complexity procedures such as free-flap creation in maxillofacial surgery, is safe and results in no increase in patient morbidity or mortality (34–40).

An attempt was made to limit potential confounding factors including the difficulty of any individual procedure vs. another. These factors were limited by including anesthetic time as a representation of the relative difficulty or technicality of each procedure. The incidence of overall complication rate or the complication rate at each time point described as influenced by anesthetic time was not statistically significant. Assuming that duration of anesthesia is representative of the difficulty of a procedure, these findings further support our hypothesis that the surgical environment does not strongly influence the rate of complications regardless of the specific procedure performed. The authors also accounted for the fact that surgical techniques and instruments available over the study period have changed and thus, a logistical regression model controlling for the surgical date (by year) was used.

The retrospective nature of this study carries inherent limitations, in the accurate interpretation and representation of medical records. Firstly, only a single post-operative recheck appointment a minimum of 7 days following the surgery with this institution or the referring veterinarian was required to be included in this study. When available, referring veterinarians' records were searched for follow-up examinations. While many patients had follow-up information available for years past their surgical date, patients without medical records outside of that single required post-operative recheck appointment were assumed to be complication free. The minimum 7-day recheck examination was meant to capture follow-up information on those patients who had immediate or early short term complications while avoiding loosing those patients who were rechecked between 7–14 days after surgery who had no complications and follow-up was not necessarily required. Secondly, patients suffering from more than one complication at any given time point were categorized based on the most severe complication. Furthermore, we recognize that patients in the major-surgical category were likely to have a wide variation in the complexity of the reparative surgery. This method of categorization may under-represent the total number of individual complications and/or the required revision treatments that these patients encountered. Finally, the nature of this study did not enable inclusion of other variables that could be considered as part of the surgical environment, including the number of people in the operatory during the procedure and the noise level in the room during the procedure. Human medical literature suggests that both of these may contribute to surgical complication rates (13, 22, 41–43), however, this information was not consistently available in the medical records and therefore not considered in the analysis reported here.

In conclusion, the dataset described in this report indicates that the incidence of complications following oromaxillofacial oncologic surgery in dogs is high. However, the majority of complications are minor and require no additional professional treatment. Up to one-third of procedures required a second surgical procedure for treatment of a major complication. This is an important finding that may have a significant impact on the client's decision making prior to pursuing treatment of an oromaxillofacial tumor. Oromaxillofacial surgery performed by residents-in-training within a veterinary teaching environment with adequate supervision appears to be safe. Finally, the timing of identified complications suggests that a minimum of 24 h of post-surgical monitoring is prudent for early detection of major complications. Given that one-quarter of the patients in this cohort had complications identified >30 days following the surgical procedure, the authors recommend a post-surgical recheck appointment >30 days following a procedure in addition to the standard 2-week post-surgical assessment. Future prospective studies investigating interactions between surgical environment and other features of oromaxillofacial tumors, such as tumor grade, surgical difficulty, and tumor margins is warranted.

## Data Availability Statement

The raw data supporting the conclusions of this article will be made available by the authors, without undue reservation.

## Author Contributions

BR: conception and design of project, acquisition, analysis and interpretation of data, manuscript drafting, and revision. KM: acquisition of data and manuscript revision. SS: analysis and interpretation of data and manuscript revision. SH: design of project, data analysis, and manuscript revision. JS: conception and design of project, analysis and interpretation of data, manuscript drafting, and revision. All authors contributed to the article and approved the submitted version.

## Conflict of Interest

The authors declare that the research was conducted in the absence of any commercial or financial relationships that could be construed as a potential conflict of interest.

## Publisher's Note

All claims expressed in this article are solely those of the authors and do not necessarily represent those of their affiliated organizations, or those of the publisher, the editors and the reviewers. Any product that may be evaluated in this article, or claim that may be made by its manufacturer, is not guaranteed or endorsed by the publisher.
